# Single Remote Sensing Image Super-Resolution with an Adaptive Joint Constraint Model

**DOI:** 10.3390/s20051276

**Published:** 2020-02-26

**Authors:** Lingli Fu, Chao Ren, Xiaohai He, Xiaohong Wu, Zhengyong Wang

**Affiliations:** 1 College of Electronics and Information Engineering, Sichuan University, Chengdu 610065, Chinahxh@scu.edu.cn (X.H.); wxh@scu.edu.cn (X.W.); wangzheny@scu.edu.cn (Z.W.); 2 Key Laboratory of Wireless Power Transmission of Ministry of Education, College of Electronics and Information Engineering, Sichuan University, Chengdu 610065, China

**Keywords:** single remote sensing image, super-resolution (SR), sparse representation, nonlocal self-similarity, local structure filter

## Abstract

Remote sensing images have been widely used in many applications. However, the resolution of the obtained remote sensing images may not meet the increasing demands for some applications. In general, the sparse representation-based super-resolution (SR) method is one of the most popular methods to solve this issue. However, traditional sparse representation SR methods do not fully exploit the complementary constraints of images. Therefore, they cannot accurately reconstruct the unknown HR images. To address this issue, we propose a novel adaptive joint constraint (AJC) based on sparse representation for the single remote sensing image SR. First, we construct a nonlocal constraint by using the nonlocal self-similarity. Second, we propose a local structure filter according to the local gradient of the image and then construct a local constraint. Next, the nonlocal and local constraints are introduced into the sparse representation-based SR framework. Finally, the parameters of the joint constraint model are selected adaptively according to the level of image noise. We utilize the alternate iteration algorithm to tackle the minimization problem in AJC. Experimental results show that the proposed method achieves good SR performance in preserving image details and significantly improves the objective evaluation indices.

## 1. Introduction

With the constant development of the remote sensing technology in recent years, many remote sensing image applications have been proposed, such as fine-grained classification [1,2], detailed land monitoring [3], and target recognition [4,5]. The performance of these applications is closely related to the image quality. However, the resolution of the remote sensing image is largely affected by the spatial resolution of the optical sensor. To improve the quality of remote sensing images, many super-resolution (SR) methods [6,7,8,9,10] have been proposed. They can be divided into multi-frame [11,12,13,14,15,16,17] and single-image SR methods [18,19,20,21,22,23].

The multi-frame SR methods aim to recover a high-resolution (HR) image from multiple low-resolution (LR) frames. A multi-frame SR method based on frequency domain was proposed in [13]. Though this method can be implemented fast, it leads to serious visual artifacts [12]. To address the above issue, many spatial domain-based multi-frame SR methods have been proposed [15,16]. Farsiu et al. [15] proposed an iterative method using $l_{1}$-norm in the fidelity and regularization terms. Patanavijitt and Jitapunkult [16] proposed a stochastic regularization term according to the Bayesian maximum a posteriori (MAP) estimation. Huang et al. [14] proposed a bidirectional recurrent convolutional network for multi-frame images SR. Although the spatial domain-based multi-frame SR methods can achieve good SR performance, they either consume quite a lot of memory or take a large amount of running time [15]. Moreover, in some cases, only one image is available instead of multi-images, which is more challenging. Therefore, single-image SR methods are significant in practical applications [23].

Generally, the problem of single-image SR can be formulated as:

(1) y=DBx+n,

where y is the observed LR image, x denotes the original HR image, B represents the blurring operator, D denotes the downsampling operator, and n is the additive Gaussian white noise with standard deviation of $\sigma_{n}$. Single-image SR methods can be roughly divided into interpolation-based prior-based methods. Interpolation-based methods [24,25] exploit adjacent pixels to estimate the unknown pixel. The interpolation-based methods have low computational complexity and can recover degraded images fast, but they may generate severe staircase artifacts and smooth out image details.

Many prior-based methods have been used to address the single-image SR problem. Tai et al. [26] exploited edge prior and single-image detail synthesis to enhance the resolution of the LR image. Some researchers have proposed example learning-based methods by exploiting the priors according to the similarity of the image. Yang et al. [22] proposed a joint sparse dictionary (HR and LR dictionaries) to reconstruct the HR image. This method assumes that the sparse vector of the HR patch is the same as the corresponding LR patch. Timofte et al. [27] proposed a joint sparse dictionary-based anchored neighborhood regression (ANR) approach, where regressors are anchored to the dictionary atoms. A joint dictionary-based statistical prediction model that assesses the statistical relationships between the sparse coefficients of LR and HR patches was introduced by Peleg and Elad [28]. NPDDR [29] trained a joint dictionary to deal with the lack of HR component and employed improved nonlocal self-similarity and local kernel to constrain the optimization process. Hou et al. [7] proposed a global joint dictionary model for the single remote sensing image SR, including the local constraint within each patch and the nonlocal constraint between HR and LR images. A single dictionary-based adaptive sparse representation scheme was presented in [30]. Dong et al. [31] proposed a single dictionary-based method, which restores the HR image by using the nonlocal self-similarity of the sparse vector. Compared with the joint dictionary methods, the single dictionary methods do not require external examples to train. Although the above methods enhance the resolution of the LR image, they are prone to generate some artifacts. Recently, deep learning technologies have been introduced into SR. For example, Dong et al. [32] proposed a deep convolutional neural network for the SR methods. Haut et al. [6] exploited a novel convolutional generator model to improve the quality of remote sensing images. Li et al. [33] applied spatial-spectral group sparsity and spectral mixture analysis to the SR problem. Tai et al. [34] proposed a deep recursive residual network(DRRN) that contains 52 convolutional layers. A generative adversarial network (GAN) for image SR methods was introduced in [35].

However, deep learning-based methods take a great deal of time to train models by using external datasets. Furthermore, they require retraining for each different degradation, which costs more training time. In contrast, the single dictionary-based sparse representation SR methods require neither external datasets nor retraining for each degradation. However, traditional single dictionary-based sparse representation SR methods ignore some complementary constraints of the image itself. Previous works show that sparse representation-based SR methods are of great significance in improving the quality of remote sensing images [7,8]. Based on the above reasons, we focus on improving the single dictionary-based sparse representation SR methods to enhance the quality of the reconstructed HR image.

Inspired by [7], we consider introducing a joint constraint for the single dictionary-based sparse representation SR methods. In the references [7,8], authors pointed out that the sparse representation model is beneficial to improve the quality of the remote sensing image. However, this kind of method does not consider the continuity and correlation of an image’s local neighborhood, and the edge preservation ability should be further improved. To address this issue, we propose a local structure constraint based on the local gradient of the image, which can further improve the edge-preserving ability. For the sparse representation-based SR methods, it is usually assumed that the sparse coefficients corresponding to the LR image and the HR image are equal. However, due to the degradation of the LR image such as blurring and down-sampling, there is a gap between the LR sparse coefficient and the HR sparse coefficient, i.e., sparse noise. In addition, image artifacts tend to appear in the reconstructed images. Since the sparse noise and image artifacts can be well suppressed by exploiting these overlapping nonlocal similar patches, as shown in [7,31], we use the nonlocal self-similarity of the image to construct a nonlocal constraint. By using the nonlocal constraint and the proposed local structure constraint, we further propose a novel adaptive joint constraint (AJC) for the single remote sensing image SR. In the reconstruction phase, according to the level of image noise, we adaptively select regularized parameters for the proposed AJC model to obtain better reconstructed images. Our main contributions are summarized as follows:

(1) Since human visual system is more sensitive to image edges, we propose a local structure filter based on the local gradient of the remote sensing image and then construct a local structural prior.

(2) The fusion of the complementary local and nonlocal priors can achieve higher SR performance. Based on this, we combine the nonlocal self-simialrity prior and the local structural prior and then propose a novel adaptive joint constraint (AJC) for the single remote sensing image SR.

(3) To further improve the proposed AJC SR method, the regularization parameters are selected adaptively according to the level of remote sensing image noise.

The organization of this paper is as follows. Section 1 is the introduction. Section 2 introduces the related work about the sparse coding-based single image SR. In Section 3, the proposed AJC algorithm is described in detail. Section 4 introduces the basic parameters setting and shows experiment results. Section 5 summarizes our work.

## 2. Related Work

The single-image SR reconstruction methods aim to transform the LR image into the HR image. To achieve this goal, sparse priors have been proposed [22,27,30,31,36,37]. Researchers have found that the image can be sparsely represented as x≈Θα, where α is a sparse vector composed of coefficients, and Θ denotes an over-complete dictionary, such as a discrete cosine transform (DCT) or a wavelet dictionary [30,38]. The sparse vector α can be evaluated via the following formula:

(2) $$\hat{\alpha }=\arg\min_{\alpha }{\{∥x-\Theta \alpha ∥}_{2}^{2}+{\iota ∥\alpha ∥}_{0}\},$$

where ι represents a regularization parameter, x is a HR image, Θ denotes the dictionary of x, and α represents the sparse vector of x. Since $l_{0}$-minimization is an NP-hard problem, in general, $l_{1}$-minimization is used to approximate the $l_{0}$-minimization. In the single-image SR problem, we want to restore the missing details of the LR image y to enhance the spatial resolution. For this problem, we can address it by the following equation:

(3) $${\hat{\alpha }}_{y}=\arg\min_{\alpha }{\{∥y-DB\Theta \alpha ∥}_{2}^{2}+\tilde{\lambda }{∥\alpha ∥}_{1}\},$$

where ${\hat{\alpha }}_{y}$ denotes the estimated sparse vector of the LR image y, and $\tilde{\lambda }$ is a regularization parameter of the sparse term. Since the sparse vector of the HR patch is the same as the corresponding LR patch [22], we can get the reconstructed HR image by $\hat{x}\approx \Theta {\hat{\alpha }}_{y}$.

## 3. Proposed Method

**Figure 1:**
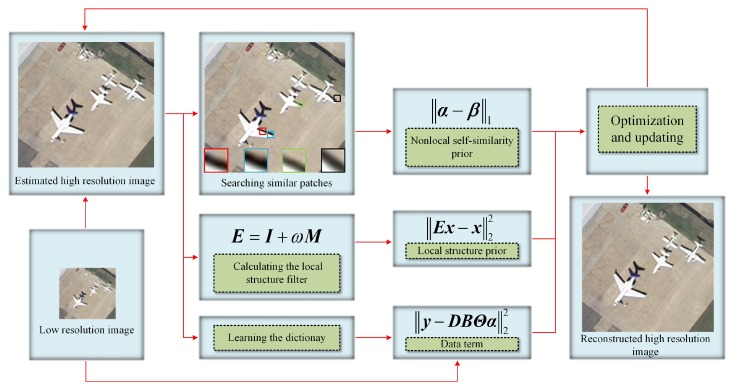
The proposed framework.

To remove artifacts and improve the quality of reconstructed HR images, we propose the AJC model that includes the nonlocal and local priors. The proposed framework is shown in Figure 1, the nonlocal prior is constructed by exploiting the nonlocal self-similarity of the sparse vector, and the local prior can be obtained according to the proposed local structure filter. The proposed AJC model can be formulated as:

(4) $${\hat{\alpha }}_{y}=\arg\min_{\alpha }\left\{{∥y-\mathrm{DB}\Theta \alpha ∥}_{2}^{2}+\eta J_{NL}\left(\alpha \right)+\gamma J_{L}\left(x\right)\right\},$$

where the first term is the data term, $J_{NL}\left(\alpha \right)$ denotes the nonlocal constraint, $J_{L}\left(x\right)$ represents the local prior, η is a parameter to balance the data term and the nonlocal constraint, and γ is a regularization parameter used to keep balance between the data term and the local structural constraint. For the subsequent reconstruction process, we first need to use the self-learning method to train the compact dictionary. Then, the nonlocal and local priors are constructed. Finally, we use the iterative shrinkage algorithm [39] to solve the SR reconstruction problem.

### 3.1. The Compact Dictionary Learning

In sparse representation, it is important to select a suitable dictionary for the image. In this paper, we learn a compact dictionary for the image according to [31]. It is worth noting that our dictionary learning uses a LR image as the input of training. The compact dictionary learning first needs to extract the HR image patches X ( For the first reconstruction, the HR image was reconstructed from the corresponding LR image by Bicubic.). Considering that human vision is sensitive to edges, we remove extremely smooth image patches in X and express them in $X^{h}$. Furthermore, to further supplement the high frequency details of the images, we learn the dictionary in the high-frequency feature space of patches. Specifically, we adopt a rotationally symmetric Laplacian of Gaussian filter (size 5×5 with standard deviation 0.5), which can improve the average PSNR of the test images by 0.11dB in SR application. The high-frequency feature of $X^{h}$ is denoted by $C=[C_{1},C_{2},\ldots ,C_{N}]$, where *N* represents the number of patches. Then, we adopt the K-means algorithm to divide C into *K* clusters $\left(H=\left[H_{1},H_{2},\ldots ,H_{K}\right]\right)$. Next, the compact dictionary Θ can be learned from H. Specially, since the PCA approach can reduce dimension and realize de-correlation, we apply the PCA algorithm to each cluster $H_{k}\left(k=1,2,\ldots ,K\right)$. After PCA, the sub-dictionary $\Theta_{k}$ corresponding to each cluster $H_{k}$ can be obtained according to [31]. Finally, the compact dictionary Θ corresponding to the reconstructed image $\hat{x}$ can be constructed by $\Theta =[\Theta_{1},\Theta_{2},\ldots ,\Theta_{K}]$. After learning the dictionary Θ, we apply the dictionary to the proposed AJC model for obtaining the reconstructed HR image $\hat{x}$. It should be noted that the estimated $\hat{x}$ will be used to update the dictionary Θ and *K* will be updated in the dictionary learning phase.

### 3.2. The Joint Priors

#### 3.2.1. The Nonlocal Self-Similarity Prior

The remote sensing image has a large number of repetitive structures. That means, for a given image patch, we can find its similar patches in other parts of the image. We illustrate the nonlocal self-similarity of the remote sensing image by using the “airplane” image in Figure 1. The red patch is a target patch, its nonlocal similar patches can be found in the image, such as the blue, green, black patches. Thus, we can use the nonlocal self-similarity of the remote sensing image to guide the image SR reconstruction. According to [31], the sparse vector also has the nonlocal self-similarity. Thus, we can exploit the nonlocal self-similarity of the sparse vector to guide the image SR reconstruction, where the nonlocal prior can be formulated as:

(5) $$J_{NL}\left(\alpha \right)={∥\alpha -\beta ∥}_{1},$$

where $\alpha =[\alpha_{1},\;\ldots ,\;\alpha_{i},\;\ldots ,\;\alpha_{N}]$, $\beta =[\beta_{1},\;\ldots ,\;\beta_{i},\;\ldots ,\;\beta_{N}]$, and $\beta_{i}$ is the estimated $\alpha_{i}$ by using the nonlocal self-similarity of $\alpha_{i}$.

In view of the above, the closer the patches are, the greater the similarity weight will be. This can be achieved by gaussian weighting. Thus, we can estimate β by the nonlocal gaussian weighting of the nonlocal similar sparse vector α. For each patch $x_{i}$, $\beta_{i}$ can be computed by the following equation:

(6) $$\beta_{i}=\sum_{x_{i}^{s}\in C_{o}}\left\{\frac{\exp(-∥x_{i}-x_{i}^{s}{∥}_{2}^{2}/\epsilon_{2})}{\sum_{x_{i}^{s}\in C_{o}}\exp(-∥x_{i}-x_{i}^{s}{∥}_{2}^{2}/\epsilon_{2})}\alpha_{i}^{s}\right\},$$

where $C_{o}$ is the collection of similar patches of $x_{i}$, $x_{i}^{s}$ denotes the *s*-th similar patch of $x_{i}$, $\epsilon_{2}$ is a constant that controls the degree of decay, and $\alpha_{i}^{s}$ represents the *s*-th similar vector of $\alpha_{i}$.

#### 3.2.2. The Local Structure Prior

However, the previous prior term can not sufficiently constrain the local structures of remote sensing images. Therefore, we propose a local prior as a complementary prior. The methods [7,22,40] exploit local features of the images to constrain the solution of SR problem. JIDM [7] and SRSC [22] employed local image patches as local features. K-SVD [40] ensured that mean square error (MSE) of each pixel neither reduction nor change in each iteration, which constrains the local structures. However, it is not sufficient for these methods to preserve edge energy while taking into account the relationship between adjacent pixels. To address the above issue, we use the local gradient of the image to solve the local structures-preserving problem in the SR reconstruction. The image gradient is sensitive to the image edge. The large gradients correspond to sharp edges of the image, while the small gradients correspond to smooth regions of the image. The objective function based on the local gradient of the image can be expressed as:

(7) $$O=\arg\min_{O}\sum_{p=1}^{Q}\left\{{\left(Z_{p}-O_{p}\right)}^{2}+\omega \left[a_{h,p}{\left({▽}_{h,p}\left(O\right)\right)}^{2}+a_{v,p}{\left({▽}_{v,p}\left(O\right)\right)}^{2}\right]\right\},$$

where *p* is the pixel location in the image, $Z\in R^{m\times n}$ (m×n represents the size of the image) denotes the clean image, $O\in R^{m\times n}$ represents the smoother image of Z, *Q*(Q=m×n) is the number of pixels in the image, $a_{h,p}$ and $a_{v,p}$ are weight vectors, ${\left({▽}_{h,p}\left(O\right)\right)}^{2}$ and ${\left({▽}_{v,p}\left(O\right)\right)}^{2}$ are the square of gradients along the horizontal and the vertical directions at the *p*-th position of the image O, respectively. In Equation (7), the first term is the data term to ensure that the image O is close to the image Z, the second term is a regularization term that makes the gradient of O as small as possible (except where the image has significant gradients) and constrains the edge energy, and the parameter ω balances the data term and the regularization term.

By using matrix notations, we can rewrite Equation (7) as:

(8) $$O=\arg\min_{O}\left\{{\left(Z-O\right)}^{T}\left(Z-O\right)+\omega \left(O^{T}S_{h}^{T}A_{h}S_{h}O+O^{T}S_{v}^{T}A_{v}S_{v}O\right)\right\},$$

where $A_{h},A_{v}\in R^{Q\times Q}$,

(9) $$\begin{array}{ccc} A_{h}= & \left[\begin{array}{cccc} a_{h,1} & 0 & \ldots & 0\\ 0 & a_{h,2} & \ldots & 0\\ \vdots & \vdots & \ddots & \vdots \\ 0 & 0 & \ldots & a_{h,Q} \end{array}\right],A_{v}= & \left[\begin{array}{cccc} a_{v,1} & 0 & \ldots & 0\\ 0 & a_{v,2} & \ldots & 0\\ \vdots & \vdots & \ddots & \vdots \\ 0 & 0 & \ldots & a_{v,Q} \end{array}\right]. \end{array}$$

**Figure 2:**
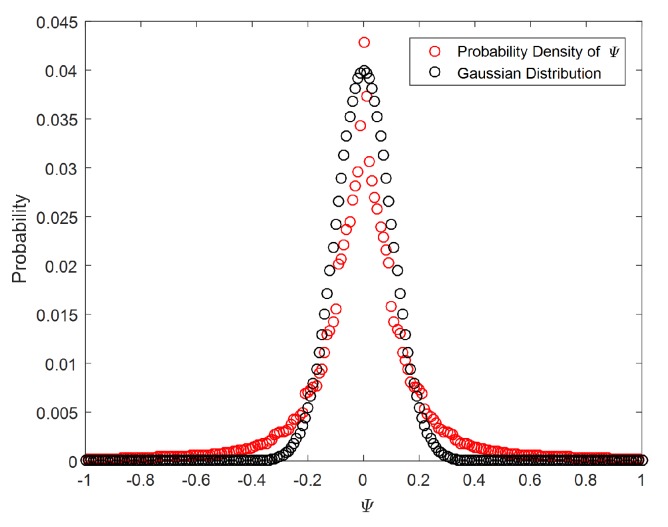
The statistic property of ψ.

$S_{h}$ and $S_{v}\in R^{Q\times Q}$ are discrete difference operations along the *h* and *v* directions, respectively, which are defined as follows:

(10) $$\begin{array}{ccc} S_{h}cr= & \left\{\begin{array}{cc} 1 & \mathrm{if}\;c=r\\ -1 & \mathrm{if}\;c=r_{0}+m\\ 0 & \mathrm{if}\;\mathrm{others} \end{array}\right.,S_{v}cr= & \left\{\begin{array}{cc} 1 & \mathrm{if}\;c=r\\ -1 & \mathrm{if}\;c=r_{0}+1\\ 0 & \mathrm{if}\;\mathrm{others} \end{array}\right., \end{array}$$

where *c* and *r* are the column and row numbers of $S_{h}$ and $S_{v}$, respectively, c,r= 1, 2, …, Q, and $r_{0}$ is the value of *r* in the case of r=c. Minimizing Equation (8), we can acquire the following formula:

(11) (I+ωM)O=Z,

where $M=S_{h}^{T}A_{h}S_{h}+S_{v}^{T}A_{v}S_{v}$, I denotes an identity matrix, and we call E=(I+ωM) the local structure filter. Inspired by [41], we calculate weight vectors $a_{h,p}$ and $a_{v,p}$ by:

(12) $$\begin{array}{c} a_{h,p}={\left[{▽}_{h,p}\left(O\right)+\epsilon_{1}\right]}^{-1},a_{v,p}={\left[{▽}_{v,p}\left(O\right)+\epsilon_{1}\right]}^{-1}, \end{array}$$

where $\epsilon_{1}$ is a constant for mathematical stability, and $\epsilon_{1}=0.0001$. Since O is unknown, $a_{h,p}$ and $a_{v,p}$ are estimated by exploiting the image Z. The calculation of the local structure filter E is summarized in Algorithm 1.
**Algorithm 1** The calculation of the local structure filter E.**Input: *Z*****Output: *E***1:set parameters $\epsilon_{1},\omega$ and compute $a_{h,p},a_{v,p}$ by Equation (12);2:calculate matrices $A_{h},\;A_{v}$ via Equation (9);3:calculate difference matrices $S_{h},\;S_{v}$ via Equation (10);4:compute the symmetrical matrix M by $M=S_{h}^{T}A_{h}S_{h}+S_{v}^{T}A_{v}S_{v}$;5:obtain the local structure filter E by E=(I+ωM);

The proposed local structure filter E has strong local correlation because it takes into account the local gradient of the image. Moreover, remote sensing images are usually terrain and targets images, such as airplane, harbor, parking lot, river, and mountain. These images have local correlation. To describe the local correlation of remote sensing images, we construct a local prior for remote sensing images. In order to explore the local property, we perform statistical analysis on a large number of remote sensing images. Since E can describe the local continuity of the image, we analyze the statistic property of ψ=Ex-x. For the image “Building” as an example, the distribution function curves are shown in Figure 2. We can find that the probability density of ψ is close to Gaussian distribution (note that the value of each entry in ψ is normalized to [−1, 1] in this test). According to the results, we can obtain the following formula:

(13) $$F\left(\psi \right)=\left\{\frac{1}{\sqrt{2\pi }\sigma_{h}}\exp\left(\frac{-\psi^{2}}{2\sigma_{h}^{2}}\right)\right\},$$

where F(ψ) is the the probability density of ψ, and $\sigma_{h}$ denotes a standard deviation. Then the local constraint $J_{L}\left(x\right)$ can be constructed according to ψ in the MAP estimate, and the mathematical expression is as follows:

(14) $$J_{L}\left(x\right)={∥\mathrm{Ex}-x∥}_{2}^{2}.$$

### 3.3. Regularized Parameter Settings

Proper parameters are conducive to improve the performance of SR reconstruction, so we adopt the parametric adaptive method. Since the noise level is related to the regularization parameters [23], in this paper, η and γ are both selected adaptively according to the noise level. The noise level can be calculated by [42]. Let ξ=α-β, and assume that ξ and ψ are independent. The MAP estimation considering both ξ and ψ is a multi-prior estimation. Multi-prior estimation methods have been widely used in image processing [43,44]. The MAP estimation of ξ and ψ can be expressed as:

(15) $$\begin{array}{cc} \xi ,\psi \; & =\arg\max_{\xi ,\psi }\left\{\log F(y|\xi )+\log F(\xi )+\log F(y|\psi )+\log F(\psi )\right\}. \end{array}$$

Since E is a pre-calculated matrix and ψ=Ex-x, ψ is only related to x. We can obtain F(y|ψ)=F(y|x).

F(y|ξ) and F(y|x) are generally characterized by the Gaussian distribution [43,44]:

(16) $$F\left(y|\xi \right)=F\left(y|\alpha ,\beta \right)=\frac{1}{\sqrt{2\pi }\sigma_{n}}\exp\left\{-\frac{{∥y-\mathrm{DB}\Theta \alpha ∥}_{2}^{2}}{2\sigma_{n}^{2}}\right\},$$

(17) $$F\left(y|x\right)=\frac{1}{\sqrt{2\pi }\sigma_{n}}\exp\left\{-\frac{{∥y-\mathrm{DBx}∥}_{2}^{2}}{2\sigma_{n}^{2}}\right\},$$

where ξ and β are assumed to be independent according to [31]. Since Θ is pre-calculated and x=Θα, F(y|ξ)=F(y|x).

F(ξ) can be obtained according to [31,45]:

(18) $$F\left(\xi \right)=\prod_{i}\prod_{g}\frac{1}{\sqrt{2}\sigma_{i,g}}\exp\left\{-\frac{\sqrt{2}{∥\xi_{i,g}∥}_{1}}{\sigma_{i,g}}\right\},$$

where $\xi_{i,g}$ is the *g*-th entry of $\xi_{i}$, and $\sigma_{i,g}$ represents a standard deviation of the error $\xi_{i,g}$ corresponding to the Laplace distribution.

Substituting Equations (13), (16), (17) and (18) into Equation (15), and ignoring constant terms, we can get:

(19) $$\alpha_{y}=\arg\min_{\alpha }\left\{{∥y-\mathrm{DB}\Theta \alpha ∥}_{2}^{2}+\sqrt{2}\sigma_{n}^{2}\sum_{i}\sum_{g}\frac{∥\alpha_{i,g}-\beta_{i,g}{∥}_{1}}{\sigma_{i,g}}+\sigma_{n}^{2}\frac{{∥\mathrm{Ex}-x∥}_{2}^{2}}{2\sigma_{h}^{2}}\right\},$$

where $\alpha_{i,g}$ denotes the *g*-th entry of $\alpha_{i}$, and $\beta_{i,g}$ represents the *g*-th entry of $\beta_{i}$.

Comparing Equation (4) with Equation (19), we can obtain parameters η and γ. Furthermore, to ensure mathematical stability, we add additional parameters $\epsilon_{\eta }$ and $\epsilon_{\gamma }$ in the calculation of η and γ, respectively.

(20) $$\eta =\sum_{i}\sum_{g}\frac{\sqrt{2}\sigma_{n}^{2}+\epsilon_{\eta }}{\sigma_{i,g}},\;\gamma =\left(\sigma_{n}^{2}+\epsilon_{\gamma }\right)/2\sigma_{h}^{2},$$

where $\sigma_{h}$ denotes the evaluated noise level from $\hat{x}$ during each iteration.

Finally, we apply the iterative shrinkage algorithm [39] to the AJC model, and the details of solution are given in Algorithm 2.
**Algorithm 2** Details of solution.1:initialization
(a)set the initializing parameter;(b)get initialized $\hat{x}$ by Bicubic of the input LR image;(c)estimate the noise level $\sigma_{n}$ of the input LR image;
2:outer loop: iterate on $l_{o}=1,2,\ldots ,l_{o}^{max}$
(a)update the dictionary Θ;(b)inner loop: iterate on l=1,2,…,L;
(1)obtain the noise level $\sigma_{h}$ of the reconstructed HR image ${\hat{x}}^{l}$;(2)estimate η and γ by Equation (20);(3)calculate the filter E by using Algorithm 1, where ${\hat{x}}^{l}$ as the input;(4)compute ${\hat{x}}^{\left(l+1/2\right)}={\hat{x}}^{l}+\delta \left[B^{T}D^{T}\left(y-\mathrm{DB}{\hat{x}}^{l}\right)+\gamma {\left(E-I\right)}^{T}\left(E{\hat{x}}^{l}-{\hat{x}}^{l}\right)\right]$;(5)estimate $\alpha_{i}^{\left(l+1\right)}$ according to $\alpha_{i}^{\left(l+1\right)}=S_{\varsigma }\left(\Lambda_{i}^{l}-\beta_{l}\right)+\beta_{l}$, where $\varsigma =\lambda_{1}^{l}/u$, *u* is an auxiliary parameter, and $\Lambda_{i}^{l}=\frac{\Theta^{T}B^{T}D^{T}\left(y-\mathrm{DB}\Theta \Theta_{k}^{T}R_{i}{\hat{x}}^{\left(l+1/2\right)}\right)}{u}+\Theta_{k}^{T}R_{i}{\hat{x}}^{\left(l+1/2\right)}$, where $\Theta_{k}$ denotes the corresponding sub-dictionary for the patch $R_{i}{\hat{x}}^{\left(l+1/2\right)}$;(6)if $\;\mathrm{mod}\;(l,L_{0})=0$, update $\beta_{i}$, where $L_{0}$ is constant;(7)reconstruct the HR image ${\hat{x}}^{\left(l+1\right)}$ according to
$${\hat{x}}^{\left(l+1\right)}=\Theta {\hat{\alpha }}^{\left(l+1\right)}={\left(\sum_{i=1}^{N}R_{i}^{T}R_{i}\right)}^{-1}\sum_{i=1}^{N}(R_{i}^{T}\Theta {\hat{\alpha }}_{i}^{\left(l+1\right)}),$$ where $R_{i}$ denotes the extraction matrix of ${\hat{x}}_{i}$;end of inner loop;

end of outer loop.3:obtain the reconstructed HR image $\hat{x}$.

## 4. Experimental Results

### 4.1. Experimental Setting

**Figure 3:**
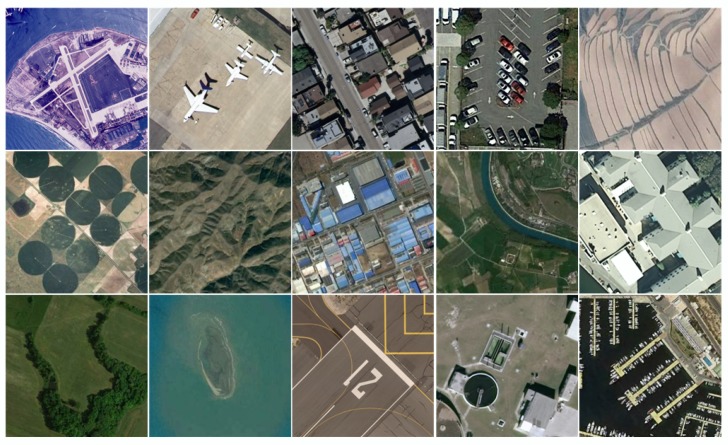
Experimental test images, including the following images: aerial, airplane, residential, parking-lot, terrace, farmland, mountain, industrial-area, river, building, meadow, island, runaway, storage-tank, harbor.

In this subsection, we show minutely the datasets and parameters settings. Test images are shown in Figure 3. These images come from three different remote sensing image datasets: UCMLUD [46], USC-SIPI-aerials [47] and NWPU-RESISC45 [48]. UCMLUD contains 21 classes of remote images, and each class contains 100 images with size 256×256. The spatial resolution for this dataset is 0.3 m/pixel. The spatial resolution for USC-SIPI-aerials is 1 m/pixel. NWPU-RESISC45 contains more kinds of images than UCMLUD. Specifically, NWPU-RESISC45 contains 45 categories, and each class has 700 images with size 256×256. The spatial resolution of this database ranges from 0.2 m/pixel to 30 m/pixel.

Furthermore, the basic parameters settings in the proposed AJC method are as follows: the number of inner loop *J* is 160, the number of outer loop *L* is 5, ω is set to 0.0001, and δ=7. In addition, to generate the test LR images, first, the HR image is blurred by the Gaussian Blur Kernel with size 7×7 and standard deviation 1.6. Then, we downsample the blurred image by a scale factor of 3 [7,49].

### 4.2. Parameters Setting

The size (τ×τ) of patch and the number (*K*) of clustering have an important impact on the SR performance. Too few clusters will eliminate the gaps between classes. Too many clusters will make the dictionary lose its representativeness and reliability. So we need to find an optimal *K* by [30]. Specifically, we first divide the training patches into *K* clusters, and then merge the classes containing a few image patches into the nearest neighboring classes. We analyze the impact of τ and $K_{0}$ on peak signal-to-noise ratio (PSNR) for all the test images, where $K_{0}$ denotes the predefined clustering number of *K*, and the results are shown in Table 1. The average PSNR varies with the patch size. In the case of the patch size of 5, the average PSNRs of the test images are superior. In the case of the same patch size and $K_{0}$ larger than 10, PSNRs are close. This phenomenon shows the robustness of the method to find an optimal *K* [30]. Furthermore, the larger the number of clustering, the more time it takes. In order to obtain a higher PSNR at a reasonable time, we set τ to 5 and $K_{0}$ to 50.

### 4.3. Comparison with Different Traditional Methods

To demonstrate the SR performance of the proposed AJC algorithm, we compare it with other SR methods, including Bicubic, SRSC [22], ASDS [30], MSEPLL [43], NARM [50] and LANR-NLM [51]. PSNR, structural similarity index (SSIM) [52] and erreur relative globale adimensionnelle de synthèse (ERGAS) [53] are used as the objective evaluation indices. The result with higher PSNR/SSIM and smaller ERGAS means the quality of the reconstructed image is better.

#### 4.3.1. Noiseless Remote Sensing Images

**Figure 4:**
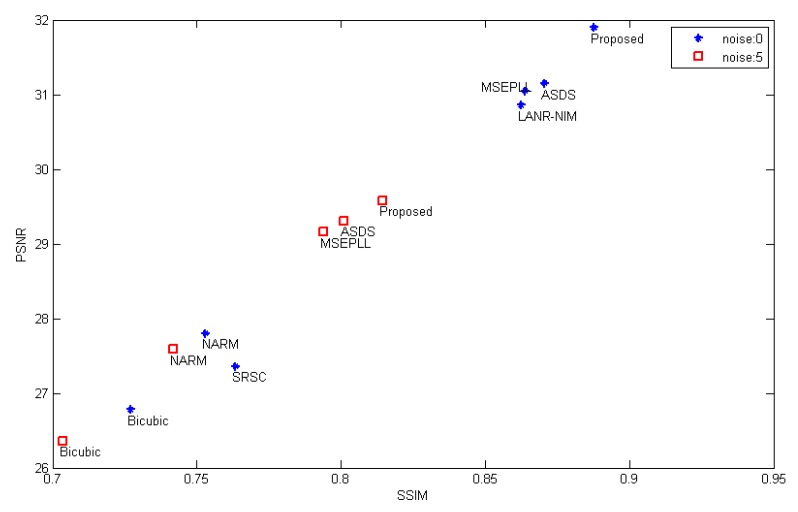
The average objective indices for the test images in the case of noise = 0 and noise = 5.

**Figure 5:**
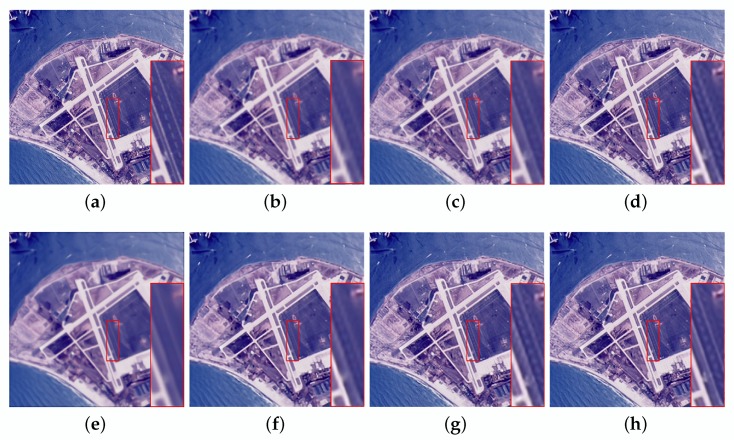
Visual comparisons of the proposed method and other methods on the aerial image (noise = 0). (**a**) original image. (**b**) Bicubic interpolation (PSNR:24.67, SSIM:0.6537, ERGAS:3.9962). (**c**) SRSC (PSNR:25.10, SSIM:0.6925, ERGAS:3.8693). (**d**) ASDS (PSNR:29.24, SSIM:0.8242, ERGAS:2.3713). (**e**) NARM (PSNR:25.97, SSIM:0.6757, ERGAS:3.6780). (**f**) MSEPLL (PSNR:29.09, SSIM:0.8138, ERGAS:2.4803). (**g**) LANR-NLM (PSNR:29.03, SSIM:0.8220, ERGAS:2.4152). (**h**) Ours (PSNR:29.61, SSIM:0.8355, ERGAS:2.2616).

**Figure 6:**
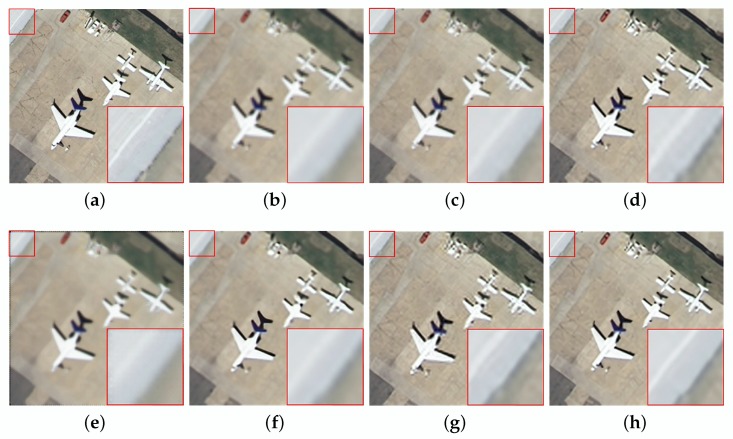
Visual comparisons of the proposed method and other methods on the airplane image (noise = 0). (**a**) original image. (**b**) Bicubic interpolation (PSNR:26.27, SSIM:0.7989, ERGAS:2.6212). (**c**) SRSC (PSNR:26.95, SSIM:0.8205, ERGAS:2.4746). (**d**) ASDS (PSNR:30.95, SSIM:0.8949, ERGAS:1.4973). (**e**) NARM (PSNR:27.20, SSIM:0.8108, ERGAS:2.6506). (**f**) MSEPLL (PSNR:31.45, SSIM:0.8944, ERGAS:1.5388). (**g**) LANR-NLM (PSNR:30.69, SSIM:0.8919, ERGAS:1.5706). (**h**) Ours (PSNR:32.19, SSIM:0.9078, ERGAS:1.3277).

In the noiseless case, the reconstruction results using different methods are shown in Table 2. For “Island”, the LANR-NLM method acquires better objective evaluation indices. However, considering all the test images, our algorithm has superior objective indices. Specifically, the average PSNR, SSIM and ERGAS are 31.90 dB, 0.8876 and 2.2912, respectively. In addition, a graph is drawn in Figure 4 for the results provided in Table 2. From Figure 4, it can be intuitively observed that our method has better objective indices than other methods. To intuitively show the visual quality of the reconstructed image, we compare the visual results as shown in Figure 5 and Figure 6. The SR performance of Bicubic interpolation is the worst. NARM produces smoother images. As shown in Figure 5f and Figure 6f, the MSEPLL method also smoothes many details of the image. In Figure 5, compare with other SR reconstructed methods, the proposed method reconstructs the HR image with fewer artifacts and clearer edges.

In Figure 6, ASDS and LANR-NLM tend to smooth out image details to some extent. As shown in Figure 6h, the image reconstructed using the proposed method has a clearer white line than others.

#### 4.3.2. Noisy Remote Sensing Images

To demonstrate the effectiveness of our method in the noisy case, we add noise with a standard deviation of 5 to the degraded image. The results are shown in Table 3.

**Figure 7:**
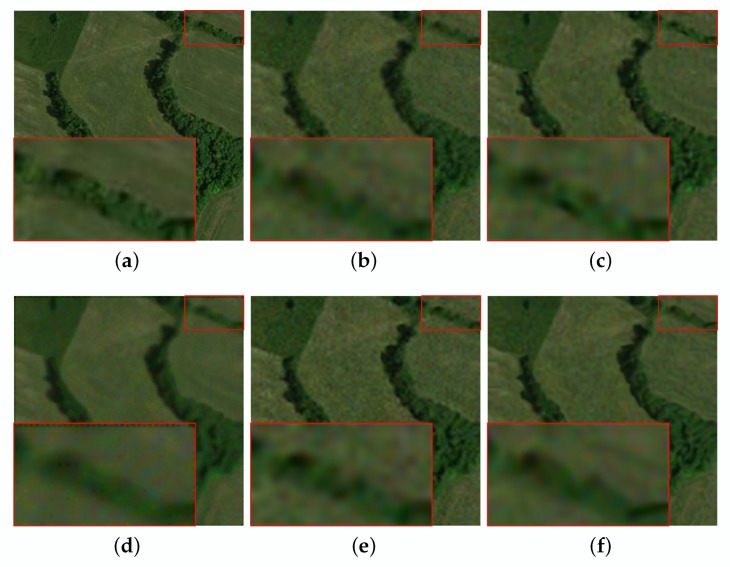
The reconstruction for the meadow of noisy image with different methods (noise = 5). (**a**) original image. (**b**) bicubic interpolation (PSNR:32.19, SSIM:0.7914, ERGAS:2.6744). (**c**) ASDS (PSNR:33.39, SSIM:0.8274, ERGAS:2.3056). (**d**) NARM (PSNR:32.56, SSIM:0.7993, ERGAS:2.7643). (**e**) MSEPLL (PSNR:33.42, SSIM:0.8260, ERGAS:/2.2176). (**f**) ours (PSNR:33.65, SSIM:0.8302, ERGAS:2.2605).

**Figure 8:**
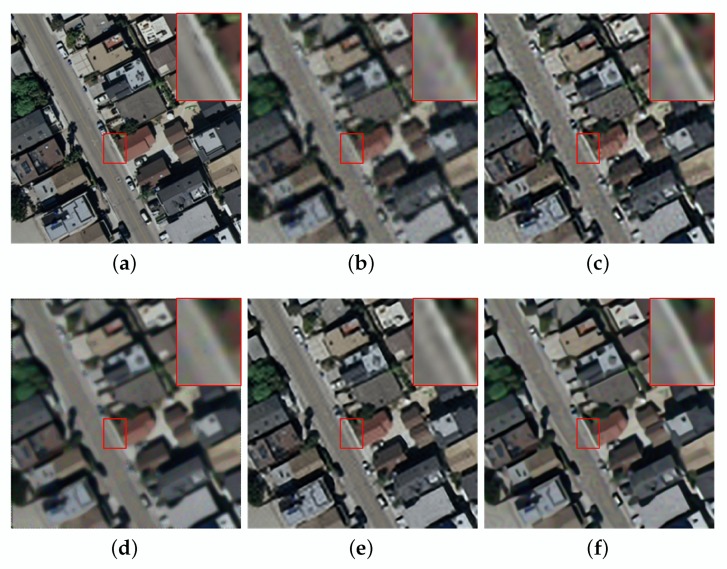
The reconstruction for the residential of noisy image with different methods (noise = 5). (**a**) original image. (**b**) bicubic interpolation (PSNR:22.05, SSIM:0.6359, ERGAS:6.6547). (**c**) ASDS (PSNR:26.16, SSIM:0.7958, ERGAS:4.1301). (**d**) NARM (PSNR:23.61, SSIM:0.6981, ERGAS:5.6835). (**e**) MSEPLL (PSNR:26.21, SSIM:0.8071, ERGAS:4.0817). (**f**) ours (PSNR:26.47, SSIM:0.8209, ERGAS:3.9976).

Compared with other methods, the ASDS method achieves better SR results on the “Aerial” image. However, the average PSNR, SSIM and ERGAS of our algorithm are the highest among these SR methods, which are 29.58 dB, 0.8144 and 2.8257, respectively. In addition, to more intuitively reflect the performance of our method, we draw a graph for the results provided in Table 3, as shown in Figure 4. Furthermore, we give visual effects as shown in Figure 7 and Figure 8. We can find that the proposed method also has better SR performance in suppressing image noise and preserving details and edges.

Consequently, according to the experiments on test images, the proposed method can achieve the best SR results in both noiseless and noisy cases.

### 4.4. Comparison with Different Deep Learning Methods

To reasonably compare with deep learning methods, we chose the classic deep learning methods SRCNN [32] and SRGAN [35], and the recently proposed remote sensing images method LGCnet [54]. For these methods, we retrain and fine tune their models, and then use these models for SR reconstruction. The training data come from three remote sensing image datasets: UCMLUD [46], USC-SIPI-aerials [47] and NWPU-RESISC45 [48]. All the training images are first blurred and then down-sampled to obtain the low-resolution images. Next, both the obtained low-resolution images and their original versions are collected as training pairs to train SRCNN [32], SRGAN [35], and LGCnet [54]. When the training error stops decreasing, we reduce their initial learning rates to fine-tuned their models to achieve their best performance. The results are shown in Table 4. For “Runway”, the resolution reconstructed by the LGCnet method is higher. However, for all the test images, our method is superior to SRCNN, SRGAN and LGCnet, and the average PSNR/SSIM gains over SRCNN, SRGAN and LGCnet are 0.84 dB/0.0215, 1.64 dB/0.0778 and 0.2 dB/0.0048, respectively. For ERGAS, our method is 0.0394 better than SRGAN on the test images. Our average ERGAS is 0.04 better than LGCnet’s average ERGAS and 0.1984 better than the average ERGAS of SRCNN. In order to show that our method does not need a lot of external data compared with the deep learning methods, we provide the training time and the number of training images, as shown in Table 5. Compared with other methods, the proposed approach can train with only one image, and saves lots of training time. The experiment results show that our method has better SR performance in noiseless cases.

### 4.5. Comparison with Different Methods on Datasets

To verify our method performance, we use different methods to reconstruct the test databases, including the “Airplane” and “Storage-tank” sub-databases from the UCMLUD, and the “Island” sub-database from the NWPU-RESISC45. The results are shown in Table 6. In Table 6, in addition to our approach, ASDS is the best traditional method, LGCnet is superior to SRCNN in deep learning methods. For the “Airplane” sub-database, our method is superior to the ASDS and LGCnet methods, and the average PSNR/SSIM gains over ASDS and LGCnet are 0.81 dB/0.0106 and 0.32 dB/0.0026, respectively. Meanwhile, our method has a better ERGAS than other methods, it is 1.7553. For the “Storage-tank” sub-database, the PSNR/SSIM of our algorithm is 0.56 dB/0.0119 higher than ASDS. Our ERGAS is 0.1194 better than the ERGAS of ASDS. Compare with LGCnet, although LGCnet has better SSIM and ERGAS, our approach gets the larger PSNR. For the “Island” sub-database, the PSNR/SSIM of our method is 0.34 dB/0.0046 superior to ASDS, and the PSNR/SSIM of LGCnet is 0.08 dB/0.0019 inferior to ours. The results show that the images reconstructed using our method has better performance.

The previous experimental analysis shows the comprehensive performance of our proposed method. Specifically, for images that satisfy the joint constraint (i.e., images with repeated structures and many edges), the proposed method has superior performance. For images with highly complex texture, the proposed method is not effective enough. In our paper, the proposed approach is based on local gradient constraint and nonlocal similarity. Because images with repeated structures and many edges can perfectly satisfy these two features, they can acquire superior reconstruction performance. Thus, in most cases, this approach performs better than many other super-resolution methods. However, for images with highly complex texture, these two features are not reliable. If we use these two features for reconstruction, the reconstructed image will not be very good. In fact, many SR approaches do not work well under such extreme conditions.

### 4.6. The Effectiveness of Joint Constraint

The image itself comprises repetitive structures, i.e., self-similarity. Researchers often exploit the nonlocal self-similarity of images for the SR reconstruction. However, it is not enough to consider only the nonlocal self-similarity to improve the resolution of the input image. To address the above problem, we construct a local constraint according to the proposed filter. Considering the joint constraint, the quality of the reconstructed image will be greatly improved. In order to demonstrate the performance of the joint constraint, we compare the SR performance for the joint constraint and the single nonlocal constraint. The results are shown in Table 7. For all the test images, the method for the joint constraint achieves superior objective qualities. Specifically, the average PSNR and SSIM gains of the joint constraint over the single nonlocal constraint are 0.38 dB and 0.0071, respectively, and the average ERGAS of the joint constraint is 0.097 better than the single nonlocal constraint. Experiment results demonstrate that the complementary joint constraint can effectively improve the image quality.

### 4.7. The Effectiveness of Adaptive Parameters

**Figure 9:**
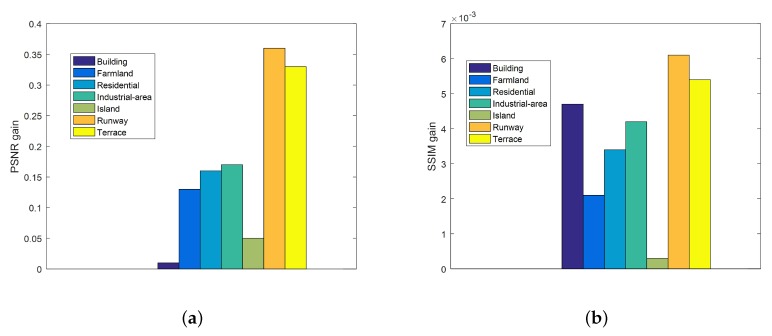
Peak Signal-to-noise Ratio(PSNR) and Structural Similarity Index(SSIM) gains of the method with adaptive parameters over the method with fixed parameters. (**a**) PSNR gains. (**b**) SSIM gains.

In the classic sparse coding problem, the choice of regularization parameters is very important. However, the parameters of most methods are fixed. In this paper, we adaptively select the parameters according to the noise level. To demonstrate the effectiveness of our adaptive parameters, we compare the results of the fixed parameters with those of the adaptive parameters as shown in Figure 9. In the case of fixed parameters, $\lambda_{1}$ and $\lambda_{2}$ are set to 0.33 and 0.001, respectively. In Figure 9, the maximum PSNR gain is 0.36 dB, and the corresponding image is “Runway”. At the same time, the image with the largest SSIM gain is also “Runway”. Specifically, it is 0.0061. For “Runway”, the ERGAS with adaptive parameters is 0.0408 better than that with fixed parameters. Experiment results indicate that the adaptive parameters are beneficial to improve the SR performance.

### 4.8. Complexity Analysis

Our method takes major cost on three part: the sub-dictionaries learning, the calculation of local structural filter, and the nonlocal search. In the sub-dictionaries learning, the core procedure involves the clustering of K-means. Its computational complexity is $O(L\tau^{2}mnK)$. The calculation of local structural filter E takes O(LJmn). The nonlocal search is related to the patch size, the search window size b×b and the number of the images. It takes $O(mn\tau^{2}b^{2})$. Take the “Airplane” with size 256×256 as a test image, the LANR-NLM method takes 40.82 s, LGCnet takes 0.74s, NARM and ASDS take 68.02 s and 107.92 s, respectively, the MSEPLL and our methods take 215.88 s and 297.91 s, respectively. Therein, traditional SR methods are implemented in MATLAB 2014a on a computer with Intel(R) Core(TM) i7-7700K CPU @ 4.20GHZ 4.20GHZ, 16.0GB RAM and 64-bit Windows 7 operating system. Our method takes a little longer time than others. This is because the proposed method learns the dictionary online for the input image and performs adaptive filtering for each iteration of the HR image. However, our method does not need external training examples, which saves a large amount of training time. In conclusion, our approach achieves the best visual and objective quality with reasonable running time.

## 5. Conclusions

In this paper, we propose a novel SR scheme based on sparse representation for the single remote sensing image. First, we use the single-dictionary method to learn the compact dictionary that exploits only the unique information of remote sensing image itself. Compared with the double-dictionary method and the deep learning method, this method has an advantage in the absence of external samples. Second, we propose a local structure filter based on the local gradient of image, and then a local structure prior is constructed. After that, the joint prior is constructed, including the local structure prior and the nonlocal self-similarity prior, which can effectively improve the fine structures recovery ability. Finally, the reconstructed HR images can be obtained by using the iterative shrinkage algorithm. The results show that the proposed local structure prior shows superior edge-preserving performance and the complementary prior constructed is more conducive in improving the SR performance. Compared with other methods, the HR images reconstructed by our scheme have better visual quality and higher objective evaluation indices. In the future, we will extend the proposed method to other image processing applications.

## Figures and Tables

**Table 1:** Average PSNR (Peak Signal-to-noise Ratio) of Test Images with Different Parameters (Scale Factor = 3, Noise = 0).

τ	$K_{0}$					
	10	30	50	70	90	110
3	31.17	31.22	31.21	31.21	31.21	31.21
5	31.84	31.86	31.90	31.87	31.87	31.86
7	31.68	31.77	31.78	31.77	31.78	31.78
9	31.49	31.57	31.59	31.58	31.59	31.59

**Table 2:** PSNR, SSIM (Structural Similarity Index) and ERGAS (Erreur Relative Globale Adimensionnelle De synthèse) Results of Reconstruction for Test Images (Scale Factor = 3, Noise = 0).

Image	Bicubic	SRSC	ASDS	NARM	MSEPLL	LANR-NLM	Proposed Method
Aerial	24.67	25.10	29.24	25.97	29.09	29.03	**29.61**
	0.6537	0.6925	0.8242	0.6757	0.8138	0.8220	**0.8355**
	3.9962	3.8693	2.3713	3.6780	2.4803	2.4152	**2.2616**
Airplane	26.27	26.95	30.95	27.20	31.45	30.69	**32.19**
	0.7989	0.8205	0.8949	0.8108	0.8944	0.8919	**0.9078**
	2.6212	2.4746	1.4973	2.6506	1.5388	1.5706	**1.3277**
Building	22.37	23.06	28.38	23.80	28.33	27.73	**29.69**
	0.7016	0.7534	0.8958	0.7478	0.8813	0.8736	**0.9295**
	4.1124	3.9017	2.0649	3.7222	2.2919	2.1973	**1.7704**
Farmland	28.35	28.72	33.50	29.84	33.74	33.39	**34.41**
	0.7874	0.8080	0.8968	0.8021	0.8940	0.8957	**0.9088**
	2.7081	2.6418	1.4812	2.5845	1.4699	1.5164	**1.3482**
Residential	22.12	22.35	27.26	23.69	27.34	26.69	**28.13**
	0.6490	0.6797	0.8612	0.7079	0.8632	0.8421	**0.8866**
	6.6038	6.4314	3.6263	5.6736	3.6207	3.9071	**3.3038**
Harbor	19.46	19.80	22.46	20.33	22.30	22.20	**22.74**
	0.5839	0.6282	0.7774	0.6250	0.7575	0.7556	**0.7954**
	9.7545	9.5490	6.9617	9.0384	7.0954	7.1817	**6.6975**
Industrial-area	24.36	24.77	28.20	25.34	28.02	27.80	**28.77**
	0.5826	0.6287	0.7951	0.6225	0.7870	0.7818	**0.8154**
	4.1738	4.0708	2.6768	3.9101	2.7803	2.7963	**2.5078**
Island	42.05	42.46	44.55	41.76	44.04	**45.54**	45.28
	0.9651	0.9678	0.9775	0.9611	0.9755	**0.9821**	0.9798
	0.6397	0.6282	0.4730	1.2450	0.4727	**0.4299**	0.4444
Meadow	32.81	33.14	35.66	32.89	35.72	35.82	**35.94**
	0.8231	0.8391	0.8960	0.8134	0.9005	**0.9016**	0.8994
	2.4863	2.4410	1.7647	2.6757	**1.6946**	1.7657	1.7365
Mountain	28.65	28.92	34.49	30.26	34.56	34.38	**34.81**
	0.7410	0.7677	0.8996	0.7660	0.8975	0.9022	**0.9063**
	3.2698	3.2337	1.6807	3.0163	1.7413	1.6866	**1.6089**
Parking-lot	20.44	20.94	24.73	21.68	24.51	24.19	**25.06**
	0.5741	0.6324	0.8019	0.6212	0.7805	0.7831	**0.8225**
	7.9069	7.6570	4.9041	7.1071	5.1441	5.1708	**4.6497**
River	28.41	28.89	31.81	28.79	31.53	31.31	**32.09**
	0.7459	0.7761	0.8710	0.7492	0.8651	0.8635	**0.8780**
	3.6894	3.5537	2.4926	3.7640	2.5060	2.6506	**2.4179**
Runway	27.08	27.44	31.94	28.47	31.56	30.93	**32.49**
	0.7765	0.7988	0.8861	0.8078	0.8726	0.8696	**0.9031**
	3.2369	3.1714	1.8489	2.9907	1.9089	2.0770	**1.7356**
Storage-tank	26.28	26.71	31.88	27.77	31.94	31.08	**33.11**
	0.8143	0.8349	0.9224	0.8353	0.9264	0.9112	**0.9367**
	3.2870	3.1918	1.6767	2.9933	1.5735	1.8949	**1.4944**
Terrace	28.32	28.93	32.15	29.27	31.58	32.13	**34.20**
	0.7066	0.7526	0.8572	0.7485	0.8464	0.8605	**0.9086**
	2.0933	2.0133	1.3215	2.2463	1.3896	1.3478	**1.0632**
Average	26.78	27.36	31.15	27.80	31.05	30.86	**31.90**
	0.7269	0.7634	0.8705	0.7530	0.8637	0.8624	**0.8876**
	4.0386	3.9257	2.4561	3.8197	2.5139	2.5739	**2.2912**

**Table 3:** PSNR/SSIM/ERGAS Results of Reconstruction for Test Images (Scale Factor = 3, Noise = 5).

Image	Bicubic	ASDS	NARM	MSEPLL	Proposed Method
Aerial	24.57/0.6385/4.0423	**27.92/0.7557/2.7563**	25.87/0.6670/3.6998	27.76/0.7388/2.8677	27.84/0.7490/2.7710
Airplane	26.13/0.7693/2.6658	29.57/0.8361/1.7637	27.06/0.7967/2.6225	29.93/0.8277/1.7034	**30.24/0.8456/1.6599**
Building	22.31/0.6817/4.1397	26.96/0.8113/2.4338	23.75/0.7405/3.7336	26.82/0.8093/2.6002	**27.27/0.8479/2.3386**
Farmland	28.09/0.7613/2.7893	31.35/0.8210/1.8997	29.67/0.7899/2.6078	31.66/0.8249/1.8446	**31.88/0.8379/1.8029**
Residential	22.05/0.6359/6.6547	26.16/0.7958/4.1301	23.61/0.6981/5.6835	26.21/0.8071/4.0817	**26.46/0.8207/4.0046**
Harbor	19.44/0.5679/9.7771	21.99/0.7214/7.3442	20.31/0.6178/8.9995	21.82/0.7063/7.4472	**22.02/0.7297/7.2726**
Industrial-area	24.26/0.5704/4.2199	26.96/0.7195/3.0857	25.21/0.6119/3.9566	26.84/0.7092/3.1590	**27.08/0.7228/3.0452**
Island	38.37/0.9164/0.9851	39.64/0.9420/0.8518	**40.32/0.9453**/1.3135	37.91/0.9068/1.0157	39.20/0.9339/**0.8952**
Meadow	32.19/0.7914/2.6744	33.39/0.8274/2.3056	32.56/0.7993/2.7643	33.42/0.8260/**2.2176**	**33.65/0.8303**/2.2597
Mountain	28.37/0.7233/3.3749	31.65/0.8168/2.3243	30.02/0.7533/3.0819	31.81/0.8196/2.3442	**32.01/0.8293/2.2192**
Parking-lot	20.39/0.5593/7.9503	**23.92**/0.7187/5.3732	21.60/0.6098/7.1449	23.82/0.7098/5.5197	23.76/**0.7283/5.3984**
River	28.16/0.7222/3.7962	30.13/0.7950/3.0248	28.64/0.7375/3.7906	29.92/0.7881/3.0064	**30.19/0.7969/3.0082**
Runway	26.90/0.7451/3.3047	29.77/0.8200/2.3667	28.44/0.7974/2.9942	29.55/0.7980/2.3862	**30.47/0.8431/2.1896**
Storage-tank	26.12/0.7854/3.3477	30.20/0.8618/2.0459	27.64/0.8227/2.9920	30.22/0.8662/1.9298	**30.50/0.8766/2.0174**
Terrace	28.07/0.6842/2.1537	30.07/0.7717/1.6848	29.10/0.7374/2.2373	29.79/0.7688/1.7139	**31.19/0.8245/1.5030**
Average	26.36/0.7035/4.1251	29.31/0.8009/2.8927	27.59/0.7417/3.8415	29.16/0.7938/2.9225	**29.58/0.8144/2.8257**

**Table 4:** PSNR/SSIM/ERGAS Results for Test Images (Scale Factor = 3, Noise = 0).

Image	Bicubic	SRCNN	LGCnet	SRGAN	Proposed Method
Aerial	24.67/0.6537/3.9962	29.04/0.8182/2.4128	29.36/0.8287/2.3298	27.89/0.7873/2.3921	**29.61/0.8355/2.2616**
Airplane	26.27/0.7989/2.6212	31.22/0.8960/1.4769	32.00/0.9033/1.3552	30.46/0.8806/1.3694	**32.19/0.9078/1.3277**
Building	22.37/0.7016/4.1124	27.83/0.8742/2.1715	28.95/0.9121/1.9262	28.02/0.9045/1.8661	**29.69/0.9295/1.7704**
Farmland	28.35/0.7874/2.7081	33.56/0.8968/1.4867	34.03/0.9043/1.4094	32.51/0.8905/1.4265	**34.41/0.9088/1.3482**
Residential	22.12/0.6490/6.6038	27.15/0.8563/3.7059	27.71/0.8757/3.4700	26.56/0.8712/3.4248	**28.13/0.8866/3.3038**
Harbor	19.46/0.5839/9.7545	22.42/0.7738/6.9924	22.67/0.7920/6.7545	21.58/0.7891/**6.5860**	**22.74/0.7954**/6.6975
Industrial-area	24.36/0.5826/4.1738	28.05/0.7878/2.7170	28.41/0.8043/2.6159	27.03/0.8063/2.6098	**28.77/0.8154/2.5078**
Island	42.05/0.9651/0.6397	45.04/0.9797/0.4557	45.05/0.9789/0.4561	42.61/0.9702/0.5597	**45.28/0.9798/0.4444**
Meadow	32.81/0.8231/2.4863	35.78/0.8989/1.7738	35.87/0.8990/1.7524	34.35/0.8845/1.7821	**35.94/0.8994/1.7365**
Mountain	28.65/0.7410/3.2698	34.50/0.9012/1.6649	34.53/0.9034/1.6612	33.06/0.8939/1.6890	**34.81/0.9063/1.6089**
Parking-lot	20.44/0.5741/7.9069	24.97/0.7937/4.7275	**25.46**/0.8204/**4.4413**	24.27/0.8102/4.3878	25.06/**0.8225**/4.6497
River	28.41/0.7459/3.6894	31.51/0.8665/2.5905	32.01/0.8756/2.4396	30.73/0.8680/2.4351	**32.09/0.8780/2.4179**
Runway	27.08/0.7765/3.2369	30.99/0.8661/2.0623	**32.59/0.9052/1.7160**	30.67/0.8716/1.8089	32.49/0.9031/1.7356
Storage-tank	26.28/0.8143/3.2870	31.48/0.9165/1.8077	32.62/0.9300/1.5836	31.09/0.9188/1.5738	**33.11/0.9367/1.4944**
Terrace	28.32/0.7066/2.0933	32.45/0.8654/1.2990	**34.26/0.9087**/1.0559	33.05/0.9023/**1.0483**	34.20/0.9086/1.0632
Average	26.78/0.7269/4.0386	31.06/0.8661/2.4896	31.70/0.8828/2.3312	30.26/0.8098/2.3306	**31.90/0.8876/2.2912**

**Table 5:** Comparison for the number of training images and training time.

	SRCNN	LGCnet	SRGAN	Proposed Method
The number of training images	2145	2145	4290	1
Training time	2 days	6 h	8 h	60 s

**Table 6:** PSNR/SSIM/ERGAS Results on Databases (Scale Factor = 3, Noise = 0).

Dataset	Airplane	Storage-Tank	Island
Bicubic	25.05/0.7351/3.7586	25.43/0.7148/4.1085	33.49/0.8812/2.6453
ASDS	30.82/0.8665/1.9192	30.04/0.8474/2.5866	37.78/0.9357/1.4686
SRCNN	30.77/0.8640/1.9225	30.05/0.8469/2.5429	37.87/0.9373/1.4911
LANR-NLM	30.29/0.8580/2.0861	29.63/0.8388/2.6896	37.77/0.9376/1.5116
LGCnet	31.31/0.8745/1.7948	30.55/0.8622/2.3560	38.04/0.9384/1.4303
Proposed method	31.63/0.8771/1.7553	30.60/0.8593/2.4672	38.12/0.9403/1.4403

**Table 7:** PSNR/SSIM/ERGAS Results of Nonlocal and Joint constraints (Scale Factor = 3, Noise = 0).

Image	Nonlocal Constraint	Joint Constraint
Aerial	29.50/0.8342/2.2917	29.61/0.8355/2.2616
Airplane	31.44/0.9026/1.4464	32.19/0.9078/1.3277
Building	27.93/0.9014/2.1688	29.69/0.9295/1.7704
Farmland	33.65/0.8993/1.4725	34.41/0.9088/1.3482
Residential	28.06/0.8834/3.3325	28.13/0.8866/3.3038
Harbor	22.18/0.7614/7.1387	22.74/0.7954/6.6975
Industrial-area	28.72/0.8129/2.5215	28.77/0.8154/2.5078
Island	44.71/0.9758/0.4746	45.28/0.9798/0.4444
Meadow	35.94/0.8996/1.7364	35.94/0.8994/1.7365
Mountain	34.80/0.9060/1.6101	34.81/0.9063/1.6089
Parking-lot	24.95/0.8188/4.7113	25.06/0.8225/4.6497
River	32.07/0.8774/2.4221	32.09/0.8780/2.4179
Runway	31.67/0.8908/1.9085	32.49/0.9031/1.7356
Storage-tank	32.92/0.9354/1.5278	33.11/0.9367/1.4944
Terrace	34.22/0.9091/1.0603	34.20/0.9086/1.0632
Average	31.52/0.8805/2.3882	31.90/0.8876/2.2912
